# Severe tetracycline dental discoloration: Restoration with conventional feldspathic ceramic veneers. A clinical report

**DOI:** 10.4317/jced.54359

**Published:** 2017-11-01

**Authors:** Vicente Faus-Matoses, Ignacio Faus-Matoses, Esther Ruiz-Bell, Vicente J. Faus-Llácer

**Affiliations:** 1 DDS, MSc, PhD. Co-director of the Master of Restorative Dentistry and Endodontics, Department of Stomatology, Medicine and Dental School, Valencia University, Spain; 2 DDS, MSc, PhD. Professor of the Master in Orthodontics, Department of Stomatology, Medicine and Dental School, Valencia University, Spain; 3 Postgraduate Student, Valencia University, Spain; 4 MD, DDS, PhD. Director of the Master of Restorative Dentistry and Endodontics, Department of Stomatology, Medicine and Dental School, Valencia University, Spain

## Abstract

The treatment of severe tetracycline discoloration is a challenge for the dentist. There are several treatment options and one of the most effective is the use of feldspathic veneers. The aim of this clinical case was to carry out the replacement with conventional feldspathic ceramic veneers on a patient with highly stained teeth (grade IV tetracycline) by a minimally invasive approach. Clinical examination revealed gingival retraction, color change of the veneers, ceramic cracked of 1.1 and 1.3 and incisal wear in lower anterior teeth. The decision was to retreat with feldspathic ceramic veneers from 1.6 to 2.6. Premolars and first molars were included because its dark color intensifies the width buccal corridors. Also, the restoration with veneers from 3.3 to 4.3 was considered. Firstly, the veneers were removed and the color was selected by the Vitapan 3D Master guide (Vita Zahnfabrick, Bad Säckingen, Germany). Mock-up was made to evaluate aesthetic, phonetic and functional parameters. Then, teeth were prepared through the mock-up in order to reduce the minimal amount of dental tissue possible. Opaque dentine mass of microhybrid composite (Esthet-X A2O, Dentsply Sirona, Konstanz, Germany) was placed to mask the most intense stains. The definitive impressions were taken with silicone (Aquasil Ultra XLV, Dentsply Sirona, Konstanz, Germany) were taken to fabricate conventional feldsphatic ceramic veneers (Noritake kisai Co, Ltd. Japan) by the parallel stratification masking technique. For cementing, an opaque cement was selected from the try-in tests (Calibra, Dentsply Sirona, Konstanz, Germany) and the composite was silicatized with an air-abrasion device. A favorable and minimally invasive result was achieved to treat grade IV tetracycline stains, surpassing the patient’s aesthetic expectations and functional needs.

** Key words:**Porcelain veneer, tetracycline teeth, minimally invasive, masking, opaque composite.

## Introduction

Tetracyclines are a group of broad-spectrum antibiotics originally found in the Streptomyces bacteria and used in treating many common infections. This antibiotic is deposited within the forming teeth and intrinsic staining may result (1).

Depending on the severity of discoloration, there are several treatment options including vital and nonvital bleaching, micro abrasion, composite and porcelain veneers, porcelain crowns and sometimes a combination of them (2).

The vital bleaching procedures cannot satisfactorily remove dark tetracycline staining. An alternative aggressive method of treatment is elective endodontic therapy with internal bleaching. Though the results are dramatic, the purposeful removal of healthy pulps is considered too radical despite the cosmetic improvement (1).

For decades, severe intrinsic staining has been often treated with crowns, but this supposes the aggressive elimination of dental structure (1,3). Currently, several types of all-ceramic systems have been developed to satisfy patients’ aesthetic demands (4).

With advancements in technology during the last decade, porcelain veneers have become a popular mean of correcting these severe problems because of the conservative amount of tooth structure that needs to be removed (3,5). Ceramic materials can be divided into two groups: based on silica (feldspathic and glass ceramic) and polycrystalline ceramics (alumina and zirconia) (4). Silica-based materials are characterized by etchability and translucency and ensure optimal aesthetics, a natural appearance, and reliable clinical performances. Conversely, polycrystalline ceramics are characterized by outstanding mechanical properties, but it is difficult to imitate the natural aesthetics of the teeth (4,6). In addition, some of the common failures of zirconia are the chipping and cracked of the veneering porcelain (6).

The feldspathic veneers present limitations to neutralize severe staining, thus the additional removal of dental structure was proposed for the placement of an opaque composite sublayer. In recent years, the parallel stratification masking technique with the controlled application of fluorescent porcelains has been carried out, avoiding the additional carving (7).

The objective of this clinical case was to carry out the retreatment of conventional feldspathic ceramic veneers in a patient with grade IV tetracycline stains, adopting a minimal invasive approach.

## Case Report

A 46-year-old woman came to the dental practice in order to change her veneers to improve her smile. The patient had feldspathic ceramic veneers from 1.3 to 2.3 for 28 years. Clinical examination revealed intense tetracycline discoloration (grade IV), gingival retraction, color change of the veneers, the ceramic of 1.1 and 1.3 was cracked and incisal wear in antero-inferior teeth. Radiographic exploration, extraoral and intraoral photographs were performed and preliminary impressions were made for the preparation of the study models.

Treatment plan consisted on veneers from 1.6 to 2.6. Premolars and first molars were included because its dark color intensifies the width buccal corridors. Besides, restorations with veneers from 3.3 to 4.3 were considered due to tetracycline stains and incisal wear.

**Figure 1:**
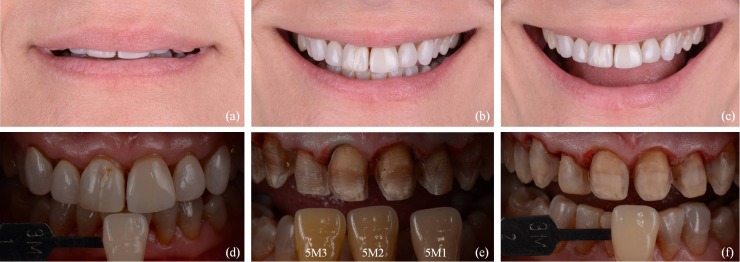
(a-c) Preoperative images: (a) lip position at rest, (b) natural smile, (c) wide smile.

**Figure 2:**
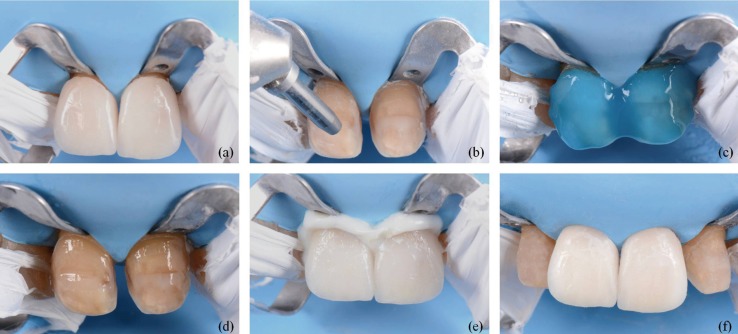
(a) Clinical try-in: feldsphatic ceramic veneers restorations in place. (b) Silicatization of the composite by silica particles and aluminum oxide. (c) Dental surface etched with 37% orthophosphoric acid. (d) Dental surface treated with universal adhesive. (e) Cementation with opaque cement. (f) Final result.

**Figure 3:**
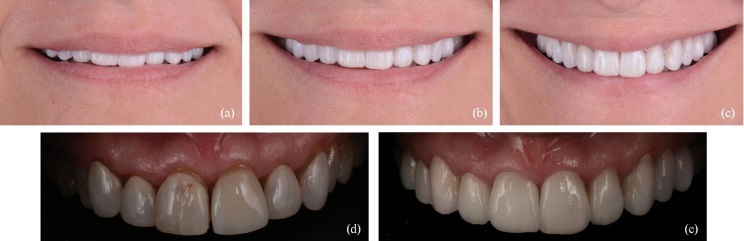
(a-c) Postoperative images: (a) lip position at rest, (b) natural smile, (c) wide smile. (d) Initial situation. (e) Final situation.

Firstly, the veneers were removed and the tooth color was selected from the Vitapan 3D Master guide (Vita Zahnfabrick, Bad Säckingen, Germany). Mock up was made to evaluate aesthetic, phonetic and functional parameters. Teeth were prepared through the mock-up in order to reduce the minimal amount of dental tissue posible. (868B 314 020, 881 314 010 y 881 314 016, Komet, Germany). Then, opaque dentine mass of microhybrid composite (Esthet-X A2O, Dentsply Sirona, Konstanz, Germany) was placed to mask the most intense stains. This technique allows the reduction of hyperchromacity and gradually increase the color value of discolored teeth (3). Afterwards, the final dental color was selected by the Vitapan 3D Master guide. The glycerine gel was placed to avoid the oxygen-inhaled layer of the composites. The definitive impressions were taken with silicone (Aquasil Ultra XLV, Dentsply Sirona, Konstanz, Germany) to fabricate conventional feldsphatic ceramic veneers (Noritake kisai Co, Ltd. Japan) by the parallel stratification masking technique. For cementing, an opaque cement was selected from the try-in tests (Calibra, Dentsply Sirona, Konstanz, Germany). The composite was silicatized by an air abrasion with a pressure of 2.5 bars from a distance of about 10 mm for 5 seconds. Next, enamel and dentin were etched with phosphoric acid (Detrey® Conditioner 36, Dentsply Sirona, Konstanz, Germany) and adhesive was applied (Prime & Bond® NT, Dentsply Sirona, Konstanz, Germany). On the other hand, the feldspathic veneers were prepared as follows: etched with 9% hydrofluoric acid for 2 minutes (Ultradent, Ultradent Products. Inc.), immersed in an ultrasonic bath with distilled water, silane (Calibra, Dentsply Sirona, Konstanz, Germany) for 2 minutes heated with a dryer and a thin layer of adhesive (Prime & Bond® NT, Dentsply Sirona, Konstanz, Germany). Finally, the cement was placed inside the veneers and placed on the teeth blandly. The palatal and buccal faces were polymerized for 40 seconds each one. The cement remnants were removed and the occlusion was checked, (Figs. 1-3).

## Discussion

In this clinical case, the patient presented intrinsic tetracycline stains (grade IV). Depending on the aetiology and severity of the stains, the treatment options ranged from a simple dental polishing to different procedures such as bleaching, veneers or crowns. The crowns are indicated as a last option to treat discolored teeth (2) because it is an aggressive treatment. In this case, the conventional feldspathic ceramic veneers were used. This restoration system is reliable and effective for the treatment of tetracycline stains (8). However, it can cause problems when the teeth are severely discolored (3). Increasing porcelain thickness improves the masking ability, but it can affect the pulp tissue (2). In addition, an excessive carving can expose dentin and the adhesion are not as predictable as in enamel (9).

We decided to use the translucent porcelain veneers combined with an opaque composite sublayer. A uniform layer of neutral dentin color along the axial surface of the tooth allows to obtain a natural result (3). Some studies affirm that the placement of opaque composite prior to the porcelain veneers can mask teeth with intense discolorations (3,8,9). Nevertheless, Bassett *et al.* (1) observed that this technique presents difficulties such as the risk of separation between opaque composite-dental surfaces prior to the cementing. Magne *et al.* (7) affirmed that the opaque resin limit is the penetration of the polymerization light. They concluded that the use of an immediate dentin sealing prior to the composite layer was necessary. An immediate dentin sealing was used for a better adhesion and to avoid the posterior bacterial filtration.

On the other hand, the bond strength between composite-porcelain may be compromised. Gresnigt *et al.* (5) carried out a clinical study to evaluate the performance of veneers united on intact teeth or teeth with composite restorations. The survival rate of the ceramic laminates bonded to teeth without (96.0%) and with existing resin composite restorations (93.5%) did not show significant differences. Chen *et al.* (8) affirm that there were not differences in the debonding rates between porcelain-composite and porcelain-tooth.

For a better result, the veneers were made by the parallel stratification masking technique. This technique allows an efficient optical integration of the restoration when the teeth are highly discolored. The first masking layer of fluorescent porcelain is applied to strongly mask the discolored areas and to create an equal base. The second masking layer is applied a little wider than the first one, as a filter to soften the transition between masking layer and natural tooth structure (7).

The use of porcelain veneers is a good treatment option due to their aesthetics properties and survival rates. The percentages described in the literature are 95.7% after 5 years (10) and 87% after 8 years (11). Katoh *et al.* (12) observed that the clinical results of the feldspathic veneers after 20 years were generally satisfactory.

Reinforced porcelain veneers also present good survival rates of 94% after 7 years (11), however, they have a lower translucency when it is necessary a high aesthetic. The alumina and zirconia are opaque materials, therefore it is difficult to achieve a good aesthetic in the anterior sector (9). In addition, they have low survival rates due to the high debonding rate, since they are non-recordable materials.

In conclusion, the placement of opaque composite prior to the placement of veneers of conventional feldspathic ceramic is a conservative treatment option for masking severe tooth discoloration.
